# Concurrent autoimmune pancreatitis and primary Biliary cirrhosis: a rare case report and literature review

**DOI:** 10.1186/1471-230X-14-10

**Published:** 2014-01-10

**Authors:** Aiqing Li, Yongjie Wang, Zheng Deng

**Affiliations:** 1 Department of Gastroenterology, Sir Run Run Shaw Hospital Affiliated to Medical School, Zhejiang University, Hangzhou, Zhejiang Province 310016, China

**Keywords:** Autoimmune pancreatitis, Primary biliary cirrhosis, Steroid, Ursodeoxycholic acid, Infection

## Abstract

**Background:**

Both autoimmune pancreatitis (AIP) and primary biliary cirrhosis (PBC) are related to various diseases. But the concurrence of AIP and PBC is extremely rare, with only 2 cases reported. Here we report the concurrence of AIP and PBC in a Chinese patient for the first time.

**Case presentation:**

A 65-year-old male was admitted to our hospital with jaundice, pruritus, mild abdominal pain and darkening urine. Serum alkaline phosphatase, γ-glutamyltransferase, bilirubin and IgG4 were prominently elevated. The antimitochondrial antibody was positive. Radiological examination revealed diffusive enlargement of the pancreas. Pancreatic biopsy showed lymphoplasmacytic infiltration, fibrosis and abundant IgG4+ plasma cells. The patient was diagnosed with AIP and PBC. Nasobiliary tube was placed to facilitate biliary drainage. A combination therapy of steroid and UDCA was administered and the patient was gradually recovered, during which the patient was complicated with biliary infecion, herpes zoster and pulmonary abscess.

**Conclusion:**

We present this case together with literature evidence to support the concurrence of AIP and PBC, share our experience of using combination therapy with steroid and UDCA, and raise the awareness of infectious complications in immunosuppressed patients.

## Background

The term autoimmune pancreatitis (AIP) defines a group of pancreatitis characterized by obstructive jaundice, lymphoplasmacytic infiltration and fibrosis and dramatic steroid response [1]. AIP has been divided into two distinct forms, in which type I is more common in Asia and more closely related to immunoglobulin G4 (IgG4) [2,3]. Recently type I AIP has been recognized as the pancreatic manifestation of a multiorgan disorder named as IgG4-related disease [2,4].

Primary biliary cirrhosis (PBC) is an autoimmune, cholestatic liver disease that affects predominantly middle-aged women and follows a slow-progressive clinical course [5-7]. Its incidence is estimated to be between 0.7 and 49 cases per million-population per year [8]. Histologically, PBC is characterized by immune-mediated destruction of small and medium intrahepatic bile ducts, which, if left untreated, will finally lead to biliary cirrhosis [5-7].

Although both AIP and PBC are related to various diseases [8,9], their concurrence in one patient is extremely rare. Only two cases have been reported in association with sclerosing cholangitis (SC) [10,11]. Here we report a Chinese patient suffering from both AIP and PBC, complicated with various infections and treated effectively with ursodeoxycholic acid (UDCA) and steroid.

## Case presentation

A 65-year-old male was admitted to our hospital complaining of jaundice, pruritus, mild right upper abdominal pain and darkening urine. Past medical history revealed atrial fibrillation and the patient was on intermittent aspirin therapy. And the family history was negative. Physical examination revealed icteric sclera and skin, mild upper abdominal tenderness, arythmia, irregular heart sound and pulse deficit.

The laboratory test data at admission showed that the serum levels of alanine transaminase (ALT), aspartate transaminase (AST), alkaline phosphatase (ALP), γ-glutamyltransferase (γ-GT), total bilirubin (TBil) and direct bilirubin (DBil) were elevated to 79 U/L (5-45 U/L), 51 U/L (5-35 U/L), 540 U/L (30-110 U/L), 197 U/L (7-50 U/L), 408 μmol/L(0-20.5 μmol/L) and 283.7 μmol/L (0-6.8 μmol/L), respectively. Serum IgG and IgM were 3,180 mg/dL (694-1620 mg/dL) and 344 mg/dL (60-263 mg/dL), respectively. The titer of antinuclear antibody (ANA) was 1:320. Screening for antoimmune diseases revealed that the patient was positive for antimitochondrial antibody (AMA), anti-AMA-M2 and anti-Ro antibody. Common tumor markers and viral markers were all negative.

**Figure 1:**
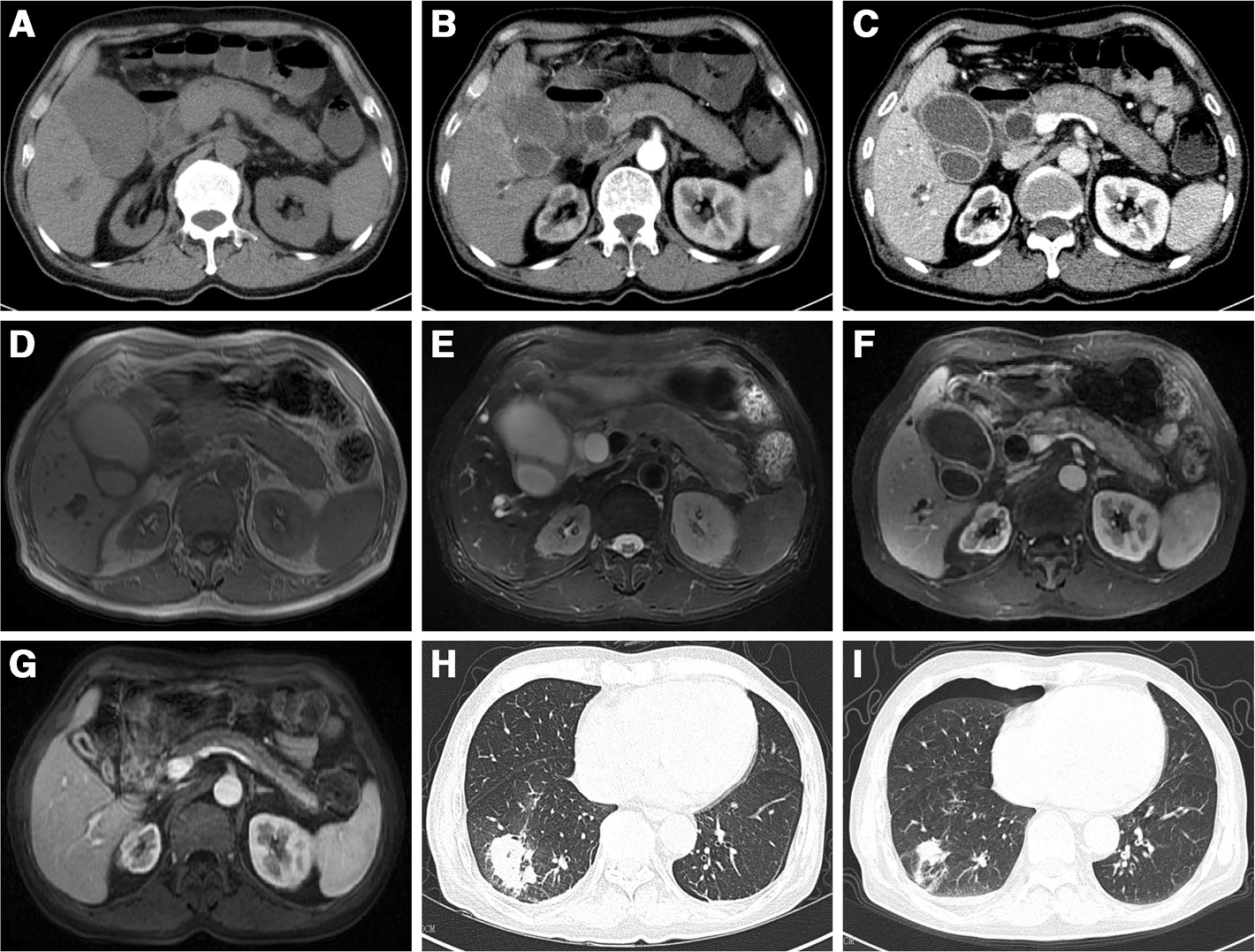
**CT and MRI scan of the patient.** Uncontract CT image **(A)**, T1 weighted image **(D)** and T2 weighted image **(E)** displayed diffusive enlargement of the pancreas capsuled by an armorlike rim, significant dilation of intra- and extra-heptic bile duct and gallbladder, and atrophy of the right kidney. Contrast CT (**B**: arterial phase; **C**: venous phase) and MRI **(F)** revealed homogeneously enhanced pancreas and diffusive thickening of the gallbladder wall. Repeat MRI **(G)** after effective treatment showed remarkable reduction of the pancreas, gallbadder and bile ducts. Chest CT **(H)** showed a large patchy shadow in the right lower lobe. Repeat CT **(I)** after antibiotic treatment showed remarkable absorption of the lesion.

Both computed tomography (CT) and magnetic resonance image (MRI) revealed diffusive enlargement of the pancreas, capsuled by an armorlike low-density rim and enhanced homogenously (Figure 1). Other abnormalities included significant dilation of intra- and extra-hepatic bile duct, diffusive thickening of the gallbladder wall and atrophy of the right kidney. Endoscopic retrograde cholangio-pancreatography (ERCP) was conducted, revealing similar findings with CT and MRI. No sign of malignancy was observed. Test of tumor markers from the bile showed CEA of 66.89 ng/mL (0-5 ng/mL) and CA199 larger than 4000 U/mL (<37.0 U/mL), but examinations of exfoliated cells proved negative for three consecutive times. The biliary culture was positive for ESBL-producing Klebsiella pneumoniae. Infection was suspected and meropenem (0.5 g per 8 hours) was administered.

**Figure 2:**
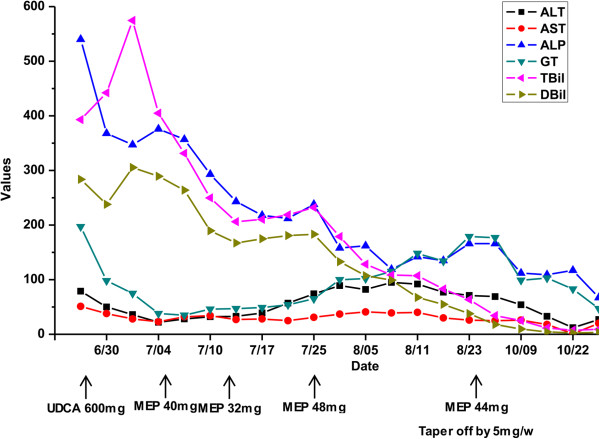
Clinical course of liver enzymes and bilirubin during combination therapy with ursodeoxycholic acid (UDCA) and methylprednisolone (MEP).

**Figure 3:**
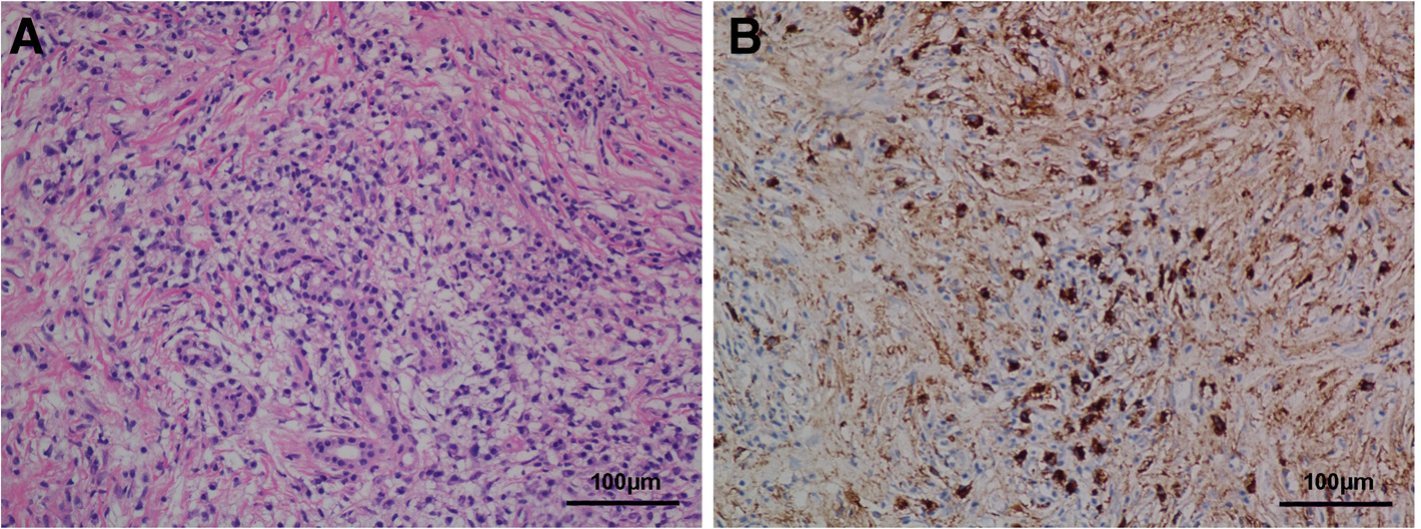
**Hematoxylin and eosin (HE) staining and immunohistochemistry (IHC) of the pancreatic tissue.** HE stain (**A**, ×200) showed dense lymphoplasmacytic infiltration and fibrosis. IHC **(B)** showed abundant IgG4+ plasma cell infiltration.

Based on the biochemical evidence of cholestasis from the elevation of ALP, γ-GT and TBil, and the presence of AMA, the patient was clinically diagnosed with PBC and UDCA (250 mg, three times per day) was initiated immediately after nasobiliary drainage [12], which partially improved the patient’s liver function (Figure 2). Since the patient was also suspected of suffering from AIP, the following examinations were conducted. IgG4 was measured and turned out to be 44.36 g/L (0.03-2 g/L). CT-guided pancreatic biopsy was performed, and histopathological evaluation showed dense lymphoplasmacytic infiltration and fibrosis. IgG4+ plasma cell was stained and counted to be as high as 95 per high-power field (HPF) (Figure 3). ECT of salivary gland was normal. So the diagnosis of type I AIP was confirmed in accordance with the diagnostic criteria [1].

Steroid therapy with intravenous methylprednisolone (MEP) (40 mg per day) was administered promptly. Serum ALP, γ-GP, TBil and DBil just before steroid therapy were 376 U/L, 38 U/L, 404.9 μmol/L and 289.2 μmol/L, respectively, and one week later were 243 U/L, 47 U/L, 47 U/L, 206.1 μmol/L and 167.1 μmol/L, respectively. Then we stepped down the steroid therapy with oral MEP 32 mg daily, yet the serum liver enzymes and bilirubin bounced up. So we increased the dose to 32 mg in the morning and 16 mg in the afternoon. And then the laboratory values improved. A repeat MRI showed remarkable reduction of the pancreas. 40 days after treatment, the IgG and IgM returned back into the normal range. The serum levels of ALP, γ-GP, TBil and DBil were 135 U/L, 134 U/L, 82.8 μmol/L and 54.9 μmol/L, respectively. The patient was discharged and followed up at the clinic with MEP tapered off weekly by 5 mg. The clinical course is depicted in Figure 2.

About 40 days after discharge, the patient developed red maculopapules on his right chest with prickling-like pain, worsened with erosion and blisters. The patient was diagnosed with herpes zoster and treated with acyclovir (500 mg twice a day) for 9 days. Unfortunately for the patient, a chest X-ray later showed multiple nodules in the right lung. Chest CT displayed multiple patchy shadows with cavity. CT-guided lung biopsy showed neutrophil aggregation and fibrous effusion within the alveoli. The patient was diagnosed with pulmonary abscess and was treated effectively with moxifloxacin (Figure 1). Given the immunosuppressed status of the patient, thymosin was injected twice a week to restore the immune function. The liver function and immunological indices of the patient were normal during the subsequent one-year follow-up period.

## Discussion

2 types of AIP have been recognized. Type I AIP commonly causes painless obstructive jaundice and frequently features elevated serum IgG4 [2,13]. Lymphoplasmacytic infiltration, storiform fibrosis, obliterative phlebitis and abundant IgG4-positive cells (>10/HPF) are the diagnostic histological changes [2,4]. In contrast, type II is more often manifested as acute pancreatitis, lacking abnormal immunological findings and sometimes associated with inflammatory bowel disease [2,13]. PBC is an autoimmune disease with uncleared etiology. It is proposed that the mitochondrial enzymes are presented to the the immune system, trigering autoantibody production and immune-mediated destruction [6,8,12,14]. Patient often presents with fatigue, pruritus, osteoporosis and hyperlipidemia [8,12,15]. Although AIP and PBC are associated with other organ involvment [8,9], their concurrence has been reported in only two cases, one being treated with UDCA and steroid [11], while the other with UDCA and stent dilation [10]. Here we report the first Chinese patient diagnosed with PBC and AIP who responded well to UDCA and steroid but was complicated with infections.

There are varying diagnostic criteria for AIP worldwide, thus making diagnosis a challenge [3]. Recently, a universal guideline has been developed based on 5 cardinal features [1], namely, characteristic radiological findings, elevated serum IgG4, other organ involvement (OOI), histopathology and response to steroid. A pancreatic biopsy is not mandatory, but necessary for patients with vague findings from CT, serology and OOI. ERCP is reserved for patients whose biopsy is unfeasible or inconclusive. Steroid trial can be considered only when cancer is excluded [2]. Given the characteritic CT and MRI features, markedly elevated IgG4 and histopathological findings, the diagnosis of AIP in our case is unambiguous.

The diagnosis of PBC is based on the biochemical evidence of cholestasis, presence of AMA and histopathology [12]. AMA is a highly disease-specific autoantibody found in 90%-95% of patients with PBC and fewer than 1% of normal controls [12], and AMA (M2) is a mitochondrial antigen specifically associated to PBC [14]. Similar to AIP, biopsy is not nessasary in typical cases unless AMA is negative [8,12]. In our case, the abnormal liver function could also be explained by AIP. Unfortunately, a premorbid test of liver funciton was not available. The patient also refused to undergo liver biopsy. A lab test 2 months after initiation of steroid therapy still showed mild cholestasis (ALP 166 U/L, γ-GT 179 U/L, TBil 63.7 μmol/L, Dbil 37.6 μmol/L), which were uncommon in AIP cases with effective biliary drainage and dramatic radiological improvement after steroid treatment. Moreover, after a thorough literature searching, we failed to find any published evidence of positive AMA in AIP. Therefore, we clinically diagnosed the patient with PBC.

Extrapancreatic lesions occur in most cases of type I AIP and are positively correlated with serum IgG4 levels [9,16,17]. The biliary system, salivary glands, thyroid, lymph nodes, lung, retroperitoneum and kidney are most frequently involved [3,18,19]. Biliary and gallbladder involvement is often manifested as diffusive wall thickening [19,20]. Renal lesions are usually shown as swelling and a nodular or an irregular pattern on CT [3,21,22]. In our case, CT image demonstrated enhancement and wall thickening of the gallbladder and common bile duct, which might be caused by obstruction due to the enlarged pancreas, or manifested as the extrapancreatic involvement in AIP. However, the right renal atrophy did not comply with the characterisitic radiological findings of IgG4 related renal lesion.

Given the systemic nature of AIP and PBC, it is tempting to think that the coexistence of AIP and PBC is not a coincidence. A survey of the pathogenesis of both diseases reveals several common features. 1. Genetic studies discovered susceptibility to both AIP and PBC conferred by HLA-DQB1 [23,24]; 2. dysregulation of cytotoxic T-lymphocyte antigen-4 (CTLA-4) was found in AIP and PBC [2,14]; 3. both AIP and PBC were most likely to be T-cell mediated [2,14]; 4. circulating autoantibodies and infiltration of plasma cells indicated an important role of B cells in AIP and PBC [2,6,25,26].

Steroid is the treatment of first choce for AIP, whose recommended starting dose is 0.6-1 mg/kg/day [1]. After 2-4 weeks, the dose is tapered by 5 mg every 1-2 weeks until it reaches 5-10 mg/day [27]. As for PBC, UDCA at a dose of 13-15 mg/kg/day is the only FDA-approved therapy [7,8,12,14]. However, UDCA does not help to relieve fatigue, pruritus, osteoporosis and concurrent autoimmune disorders [12]. Liver transplantation is reserved as the final option for end-stage liver failure [8,25]. There is limited experience in treating concurrent AIP and PBC. The combination of UDCA and steroid seems to be nessasary. Naitoh has shown that discontinuation of UDCA resulted in the elevation of liver enzymes [11]. In our case, tapering steroid off too early led to the rebounce of bilirubin levels.

In our case, the patient developed biliary infecion, herpes zoster and pulmonary abscess. Therefore, infectious complications should be noted during treatment of AIP and PBC. It was found that benign extrahepatic biliary obstruction could promote bacterial translocation [28]. Obstruction relief and infection control are the keys to preventing septic complications. Steroid use is also associated with infections [29]. Herpes zoster and uncommon pulmonary abscess have been reported [29-32]. Active immunization is recommended before applying steroid in patients without varicella zoster virus vaccination [29]. Pulmonary abscess is mainly controlled with antibiotics and postural drainage [33]. In our case, the patient was controlled effectively with antiviral drugs and antibiotics. Finally thymosin was administered to ramp up the patient’s immune function.

## Conclusion

In summary, we report an extremely rare Chinese case of concurrent AIP and PBC. Unlike previous reported cases, this patient’s clinical course was complicated by various infectious complications. The purpose of presenting this case is to provide insights into the concurrence of AIP and PBC, stress the importance of combination therapy with steroid and UDCA, and raise the awareness of infectious complications in immunosuppressed patients.

## Consent

Written informed consent has been obtained from the patient for publication of this case report and any accompanying images. A copy of the written consent is available for review by the Editor of this journal.

## Abbreviations

AIP: Autoimmune pancreatitis; IgG4: Immunoglobulin G4; PBC: Primary biliary cirrhosis; SC: Sclerosing cholangitis; UDCA: Ursodeoxycholic acid; ALT: Alanine transaminase; AST: Aspartate transaminase; ALP: Alkaline phosphatase; γ-GT: γ-glutamyltransferase; TBil: Total bilirubin; DBil: Direct bilirubin; ANA: Antinuclear antibody; AMA: Antimitochondrial antibody; CT: Computed tomography; MRI: Magnetic resonance image; ERCP: Endoscopic retrograde cholangio-pancreatography; HPF: High-power field; ICDC: International consensus diagnostic criteria; MEP: Methylprednisolone; OOI: Other organ involvement; CTLA-4: Cytotoxic T-lymphocyte antigen-4; HE: Hematoxylin and eosin; IHC: Immunohistochemistry.

## Competing interests

All authors declare no competing interest.

## Authors’ contributions

LAQ and WYJ drafted the first manuscript and made a contribution to data acquisition and interpretation. LAQ and DZ performed the clinical work-up and literature search. WYJ and DZ revised the language and grammar of the manuscript. WYJ and LAQ revised the manuscript to get final approval of the current submission. All authors read and approved the final manuscript.

## Pre-publication history

The pre-publication history for this paper can be accessed here:

http://www.biomedcentral.com/1471-230X/14/10/prepub
